# The tetraspanin web revisited by super-resolution microscopy

**DOI:** 10.1038/srep12201

**Published:** 2015-07-17

**Authors:** Malou Zuidscherwoude, Fabian Göttfert, Vera Marie E. Dunlock, Carl G. Figdor, Geert van den Bogaart, Annemiek B. van Spriel

**Affiliations:** 1 Department of Tumor Immunology, Radboud Institute for Molecular Life Sciences, Radboud University Medical Center, Nijmegen, The Netherlands; 2 Department of NanoBiophotonics, Max Planck Institute for Biophysical Chemistry, Göttingen, Germany

## Abstract

The spatial organization of membrane proteins in the plasma membrane is critical for signal transduction, cell communication and membrane trafficking. Tetraspanins organize functional higher-order protein complexes called ‘tetraspanin-enriched microdomains (TEMs)’ via interactions with partner molecules and other tetraspanins. Still, the nanoscale organization of TEMs in native plasma membranes has not been resolved. Here, we elucidated the size, density and distribution of TEMs in the plasma membrane of human B cells and dendritic cells using dual color stimulated emission depletion (STED) microscopy. We demonstrate that tetraspanins form individual nanoclusters smaller than 120 nm and quantified that a single tetraspanin CD53 cluster contains less than ten CD53 molecules. CD53 and CD37 domains were adjacent to and displayed only minor overlap with clusters containing tetraspanins CD81 or CD82. Moreover, CD53 and CD81 were found in closer proximity to their partners MHC class II and CD19 than to other tetraspanins. Although these results indicate that tetraspanin domains are adjacently positioned in the plasma membrane, they challenge the current view of the tetraspanin web of multiple tetraspanin species organized into a single domain. This study increases the molecular understanding of TEMs at the nanoscale level which is essential for comprehending tetraspanin function in cell biology.

The organization of proteins and lipids in the plasma membrane is crucial for fundamental cellular functions, including cell communication, signal transduction and trafficking. Specialized tetraspanin-enriched microdomains (TEMs) in the plasma membrane are implicated in the compartmentalization of specific lipids, receptors and signaling molecules into multi-molecular complexes^1^. Tetraspanins are small hydrophobic proteins with four transmembrane domains, a small and a large extracellular loop and two short cytoplasmic tails^2^. Tetraspanins are characterized by their ability to laterally organize membrane proteins by interacting *in cis* with transmembrane receptors, adhesion molecules, enzymes, signaling proteins and with each other. By this means, they have been proposed to organize functional TEMs in the plasma membrane that contain different tetraspanins and their interacting partner proteins^3^,^4^. The interaction of tetraspanins with their partner proteins can be direct (primary) or indirect (secondary to tetraspanin-tetraspanin interactions). The large number of different partner molecules may underlie the involvement of tetraspanins in a wide variety of essential cellular processes, including cell proliferation, differentiation, and migration^5^.

The expression profile of certain tetraspanin proteins is tissue-restricted, for example CD53 and CD37 are exclusively expressed on immune cells where they interact with various immunoreceptors^6^. In particular, many tetraspanins have been reported to associate with major histocompatibility complex (MHC) class II molecules, central receptors expressed on antigen-presenting cells (APCs; B cells, dendritic cells) that allow presentation of antigenic peptides to T cells^7^,^8^,^9^. It has been proposed that clustering of MHC class II molecules in the plasma membrane of APCs is crucial to efficiently activate T cells^10^, and the stability of MHC class II clusters on the plasma membrane may be increased by participation into microdomains, such as TEMs or lipid rafts^11^,^12^. In B cells, the interaction between tetraspanin CD81 and CD19 is crucial for cell surface expression of CD19 and B cell activation. CD81-deficiency in mice and humans leads to aberrant CD19 expression and impaired humoral immune responses indicating that tetraspanin-partner interactions are biologically relevant^13^,^14^.

The assembly of TEMs is complex and has been hypothesized to involve both tetraspanin-partner interactions and tetraspanin-tetraspanin interactions. This concept of a TEM was initially studied by biochemical approaches including isolation of detergent resistant membranes, co-immunoprecipitation, protein crosslinking and proteomics^15^,^16^,^17^. While these techniques have been instrumental to the original identification of TEMs, they do not provide insight in the spatiotemporal characteristics of TEMs in the plasma membrane. Although advanced imaging techniques have recently been applied to investigate the organization of TEMs^8^,^18^,^19^,^20^,^21^, many basic physical properties of TEMs including their size, distribution and architecture in native plasma membranes are still unknown. Given the essential role of TEMs for many important cellular processes, this is surprising. Super-resolution microscopy techniques allow to resolve individual TEMs and thereby enable the quantification of these physical properties^22^,^23^.

In this study, we visualized the tetraspanin web on the cell surface of human antigen-presenting cells using dual color stimulated emission depletion (STED) microscopy^24^,^25^,^26^. We demonstrate that tetraspanins CD37, CD53, CD81 and CD82 form individual clusters on the plasma membrane of a size below 120 nm. These small nanoclusters are distributed on the plasma membrane at densities of 1–5 domains per μm^2^. Whereas TEMs containing tetraspanin CD53 or CD81 are in proximity to their interaction partners MHC class II or CD19, these TEMs show surprisingly little overlap with other tetraspanin proteins. Finally, we quantified that a single TEM contains on average only about 3.5 molecules of CD53. Based on these data we propose a new view on the tetraspanin web.

## Results

We employed dual color STED microscopy to analyze the organization of endogenously-expressed tetraspanin proteins at super-resolution in native plasma membranes. By applying an ultrasound pulse to attached cells, we created flat membrane sheets, which allows for antibody binding of intracellular epitopes without the need for detergents^27^,^28^. Since intracellular membranes are removed, this method permits plasma membrane visualization by STED microscopy without interference by background signal arising from intracellular structures or the apical membrane. This procedure does not change the spatial localization of membrane proteins^29^,^30^, as the nanoscale distribution and size of the tetraspanin CD53 clusters in membrane sheets was comparable to those found in whole cells when we used an antibody recognizing an extracellular epitope of CD53 (Fig. S1a). The density and size of CD53 clusters were similar (whole cell: 5.01 clusters/μm^2^ and 111.5 ± 29.58 nm, membrane sheet: 5.34 clusters/μm^2^ and 102.0 ± 30.89 nm). Moreover, the cortical cytoskeleton remained at least partially intact in membrane sheets (Fig. S1b), and disruption of F-actin with Latrunculin A did not affect tetraspanin cluster distribution (Fig. S1c).

**Figure 1:**
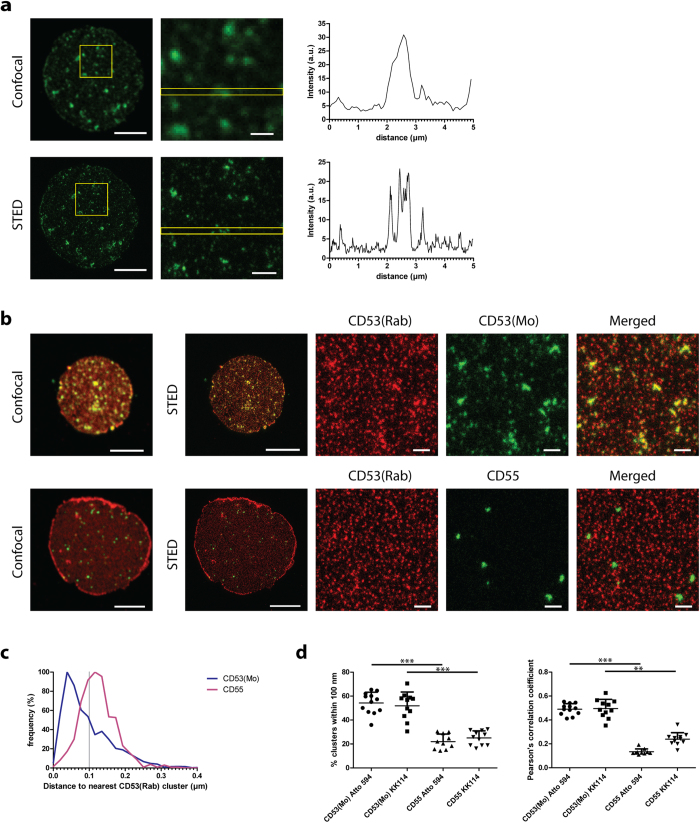
Membrane clusters enriched in tetraspanin CD53 visualized by STED microscopy. **A**: Left images: a representative JY B cell membrane sheet stained with antibodies against CD53 and imaged with conventional confocal (upper) or STED microscopy (lower). Right images: magnification of the area indicated in the left images. Scale bars represent 5 (left) and 1 μm (right). Graphs show intensity profiles of CD53 depicted in the right images. **B**: B cell membrane sheets were stained for CD53(Rab) and CD53(Mo) (upper), or CD53(Rab) and CD55 (lower) and imaged with conventional confocal microscopy or by STED microscopy. Most right image: merged image of CD53(Rab) (red) and CD53(Mo) or CD55 (green). Scale bars represent 5 μm in whole sheet images and 0.5 μm in zoomed images. **C**: Nearest neighbor analysis. Distance distributions of CD53(Mo) (blue) or CD55 (pink) clusters to the nearest CD53(Rab) clusters from at least 10 sheets. **D**: Left: For every sheet the percentage of clusters within 100 nm from the nearest CD53(Rab) cluster was plotted. ANOVA ***, significance by post hoc analysis (Bonferroni) is shown. Right: for every sheet the Pearson correlation coefficient was determined and plotted. Kruskal-Wallis ***. Significance by post hoc analysis (Dunn) is shown. CD53(Mo) Atto594 or CD55 Atto594: sheets were stained with CD53(Mo) or CD55 which was detected by anti-mouseAtto594, and CD53(Rab) was detected by anti-rabbitKK114. CD53(Mo) KK114 or CD55 KK114: secondary antibody dyes were swapped. At least 10 sheets were analyzed for each condition.

We first focused on the archetypical tetraspanin CD53 (also known as Tspan25), which is expressed exclusively in the immune system and highly abundant on antigen-presenting cells. Conventional confocal microscopy analyses revealed that CD53 was clustered on the plasma membrane of B cells, in line with the concept of tetraspanin-enriched microdomains. As the limited resolution of conventional optical microscopy does not allow to distinguish individual domains adequately, we performed STED microscopy to determine the size and distribution of individual clusters enriched in tetraspanin CD53 at the nanoscale level (Fig. 1a). With the super-resolution obtained by STED microscopy, it became apparent that many of the larger CD53 clusters observed with conventional confocal microscopy were actually multiple smaller CD53 clusters in close proximity. We employed two different CD53 antibodies in our studies; a CD53 monoclonal rabbit antibody (Rab) directed against the intracellular C-terminus of CD53, and a monoclonal mouse antibody (Mo) which recognizes the large extracellular loop of CD53. Using dual color STED microscopy, CD53 was recognized by both antibodies in clusters that clearly co-localized, showing that the antibodies bound the same target protein (Fig. 1b), in contrast to isotype control antibodies that were hardly detectable (Fig. S1d). The specificity of the CD53(Rab) antibody was confirmed in control stainings of membrane sheets of different CD53-negative cells (Fig. S1e), and the sensitivity of the two antibodies was comparable (Fig. S1f). These experiments indicate that the CD53(Mo) antibody labeled a subpopulation of CD53 proteins expressed in larger domains that were also positively stained with the CD53(Rab) antibody. This is most likely due to epitope masking by the molecular configuration in a subpopulation of CD53 molecules. As control, B cells were labeled with an antibody that recognizes the GPI-anchored protein CD55, a regulator of complement activation, which is reported not to interact with tetraspanins^31^. Indeed, CD55 labeling was found to be excluded from CD53-enriched domains (Fig. 1b).

To quantify the distance between clusters, we first annotated their positions by identifying all individual clusters in the STED images with a simple fluorescence intensity offset. The x/y coordinates of the center of each individual domain were then annotated by blob detection (Fig. S2a). We then performed a nearest neighbor analysis to calculate the distances between a CD53(Mo) or CD55 cluster to the nearest CD53(Rab) cluster. Clusters of which their center was within a distance of 100 nm from the center of the nearest CD53(Rab) cluster were considered as (partly) overlapping with CD53 (Fig. 1c). By using this criterion, 55% of clusters recognized by CD53(Mo) were overlapping with CD53(Rab) clusters. This value is an underestimate, since CD53(Mo) recognizes larger clusters than CD53(Rab) and the centers of merged CD53 clusters may therefore not always overlap. In comparison, only 22% of the CD55 clusters overlapped with CD53(Rab) clusters (Fig. 1d). To confirm these findings, we employed an alternative analysis were we calculated Pearson’s correlation coefficients for every membrane sheet. Here we obtained average values of 0.5 for CD53(Mo) and CD53(Rab), and 0.2 for CD55 and CD53(Rab) (Fig. 1d) validating the nearest neighbor analyses. Swapping the dye colors of the anti-mouse and anti-rabbit secondary antibodies did not affect the nearest neighbor distances of CD53(Mo), or CD55, to CD53(Rab) (Figs S2b and 1d). Furthermore, secondary-antibody related clustering artifacts can be excluded^32^, because similar cluster sizes and distributions of CD53 were observed by comparing whole secondary antibodies with labeled F(ab) fragments (Fig. S3a and b). F(ab) fragments contain only a single paratope, and therefore cannot induce protein clustering^28^. Moreover, we can exclude crowding effects due to the use of secondary antibodies, because the staining intensity of the secondary anti-mouse antibody correlated linearly with the intensity of the directly labeled CD53 antibody (Fig. S3c), and labeling multiple tetraspanin species did not reduce staining intensities of the individual antibodies (Fig. S3d).

### Characterization of individual tetraspanin clusters

**Figure 2:**
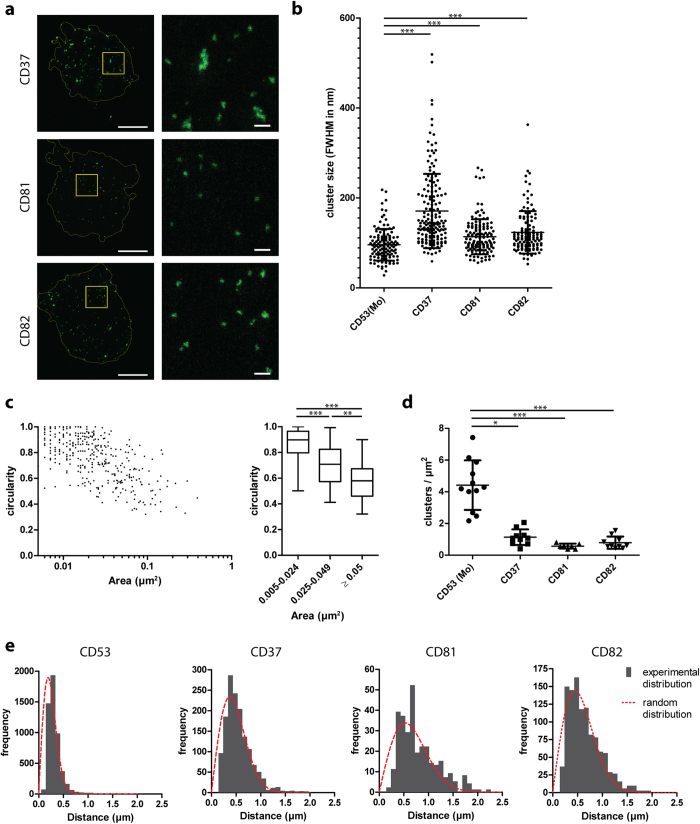
Physical characteristics of tetraspanin clusters. **A**: JY B cell membrane sheets were stained for CD37, CD81 or CD82 and imaged by STED microscopy. Right images: magnification of the area indicated in left images. Scale bars represent 5 μm in whole sheet images and 0.5 μm in zoomed images. **B**: The diameter of tetraspanin clusters measured from intensity profiles by the full-width at half-maximal intensity (FWHM). At least 120 random clusters were measured derived from at least 3 sheets per condition. Kruskal-Wallis ***. Significance by post hoc analysis is shown (Dunn’s multiple comparison tests between CD53 and other tetraspanins). **C**: Masks were made for individual CD37 clusters by applying a local threshold on STED images, and of each cluster the circularity was plotted against the area. 405 clusters were analyzed from 3 sheets. Left: dot plot of individual clusters, right: boxplot, Kruskal-Wallis ***, significance by post hoc analysis (Dunn) is shown. **D**: Density of tetraspanin clusters. For every sheet the analyzed surface area and the number of annotated clusters were determined and the number of clusters per μm^2^ calculated. At least 7 sheets were analyzed per condition. Kruskal-Wallis ***, significance by post hoc analysis (Dunn) is shown. **E**: Distances from the center of annotated clusters to the center of their nearest neighbor were determined. The red dashed curves show fits of the nearest neighbor curves with random cluster distributions and the average densities from panel D. Clusters of at least 7 sheets were analyzed per condition.

Next, we characterized the distribution and size of clusters that contain different members of the tetraspanin superfamily (CD53, CD37, CD81 and CD82) that are endogenously expressed on the plasma membrane of B cells (Fig. 2a). CD53 was present in clusters with an average size of 96 nm (±35 nm; standard deviation) (Fig. 2b). CD37-containing clusters had on average larger diameters than clusters of other members of the tetraspanin family (171 ± 82 nm). However, this was caused by a small population of relatively large domains and the majority of clusters of CD37 had sizes ranging between 100 and 150 nm, similar to the size of CD53, CD81 (114 ± 39 nm) and CD82 (124 ± 47 nm) domains. These data indicate that most tetraspanin proteins were present in clusters of a typical size of around 120 nm in the plasma membrane. This value is an upper estimate, as it is still convoluted with the resolution of our STED microscope (full-width at half-maximum intensity below 50 nm), and at super-resolution the size of the primary and secondary antibodies add to the domain sizes. Next, we determined the circularity of the tetraspanin clusters, and observed that clusters with a large area (≥0.025 μm^2^) were less circular than small clusters (Fig. 2c), suggesting that individual tetraspanin clusters can coalesce into larger clusters. Quantification of the density of tetraspanin clusters revealed that B cells contained more CD53-containing clusters on the plasma membrane than clusters containing CD37, CD81 or CD82 (Fig. 2d; on average 4.4 clusters per μm^2^ for CD53 versus approximately 0.5–1 clusters per μm^2^ for the other tetraspanins). These differences in densities of the tetraspanin clusters on the plasma membrane were in line with the expression levels of these tetraspanins as determined by flow cytometry (Fig. S4).

Next, we investigated whether the distributions of the individual tetraspanin clusters on the plasma membrane were randomly organized at the mesoscale level (i.e. relative to each other). The locations of individual tetraspanin clusters were annotated and the nearest neighbor distances to adjacent clusters (i.e. of the same tetraspanin species) were calculated. The distributions of these distances were compared to the theoretical distributions if the domains would be completely randomly distributed. The experimental distance distributions of CD53, CD37, CD81 or CD82 clusters could be well fitted with random distributions (Fig. 2e). Thus, we did not obtain evidence for a non-random mesoscale organization of clusters of individual tetraspanin species on the plasma membrane.

### Dual color STED microscopy of the tetraspanin web

**Figure 3:**
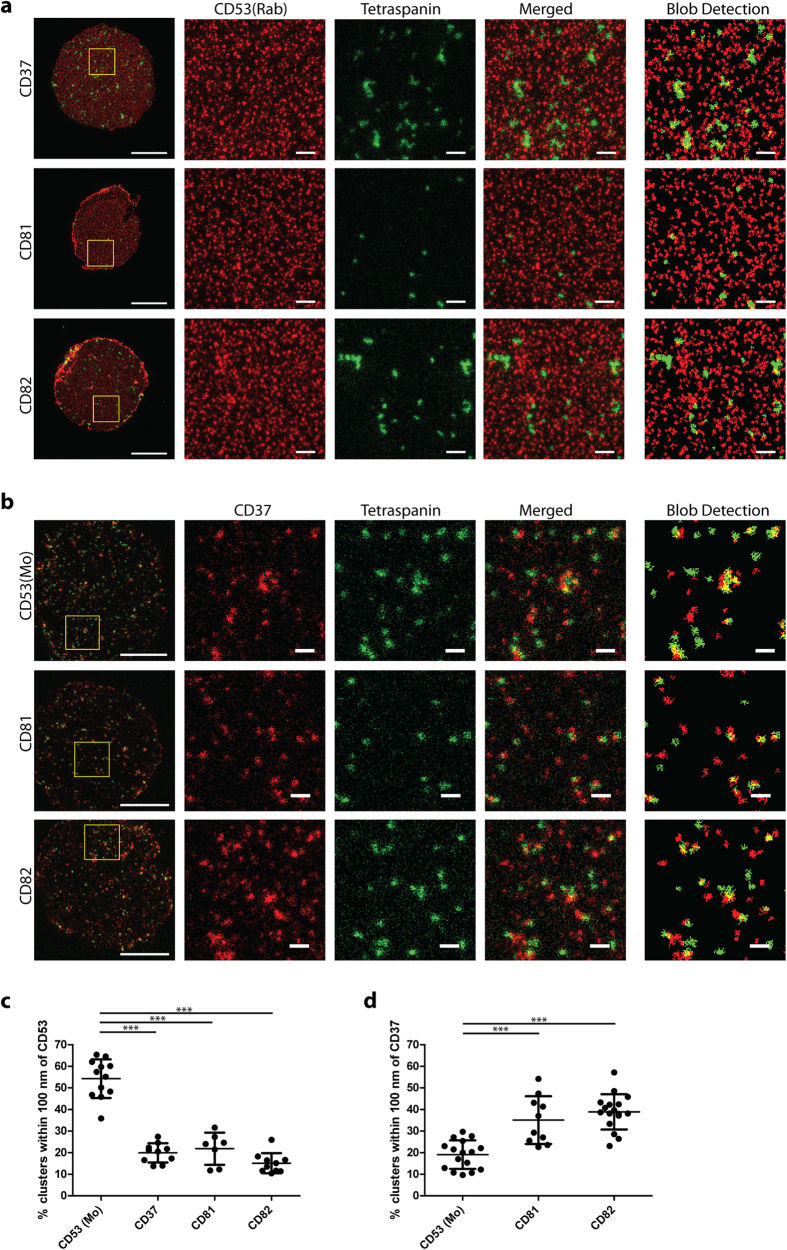
The tetraspanin web revealed by dual color STED microscopy. **A** and **B**: JY B cell membrane sheets double stained for CD53 (red) and CD37, CD81 or CD82 (green) (**A**), or double stained for CD37 (red) and CD53, CD81 or CD82 (green) (**B**) were imaged by STED microscopy. Middle images: magnification of the area indicated in whole sheets images (most left). Blob detection images (most right) show annotated clusters used for further analysis. Scale bars represent 5 μm in whole sheet images and 0.5 μm in zoomed and blob detection images. **C** and **D**: Percentage of clusters of which the distance from their center to the center of the nearest CD53 (C) or CD37 (D) cluster was within 100 nm. Clusters of at least 7 sheets were analyzed per condition. ANOVA ***, significance by post hoc analysis (Bonferroni) is shown.

Mainly based on co-immunoprecipitation studies^15^, it is widely acknowledged that tetraspanin proteins can interact with other members of the tetraspanin family. We therefore analyzed the distribution of tetraspanin clusters enriched in CD37, CD81 or CD82 relative to clusters containing the tetraspanin CD53 using dual color STED microscopy. As shown in Fig. 1b, CD53(Mo) was overlapping with CD53(Rab) clusters. Surprisingly, other members of the tetraspanin family did not co-localize with CD53 (Fig. 3a). Similarly, clusters detected by anti-CD82 rabbit antibodies (CD82(Rab)) were overlapping with clusters detected by anti-CD82 mouse antibodies, in contrast to CD82(Rab) clusters which did not co-localize with CD53(Mo) clusters (Fig. S5a and b). Next, we investigated the localization of tetraspanin CD37 clusters relative to clusters containing CD53, CD81 and CD82. Notably, CD37 clusters showed more overlap with CD81 and CD82 than with CD53 clusters (Fig. 3b). As expected, distances between clusters identified with CD53(Mo) and CD53(Rab) antibodies were shorter than distances between CD53 clusters and clusters containing other tetraspanin family members. In fact, CD53 was no closer to tetraspanins CD37, CD81 and CD82 than to CD55, a GPI-protein that is excluded from the tetraspanin web (Figs 3c and 1d). In contrast, more CD81 and CD82 clusters were within 100 nm from CD37 compared to CD53 clusters, indicating that these tetraspanins may at least partially co-cluster (Fig. 3d). These findings question the current model of tetraspanin-enriched microdomains containing multiple different members of the tetraspanin superfamily, at least for CD53.

**Figure 4:**
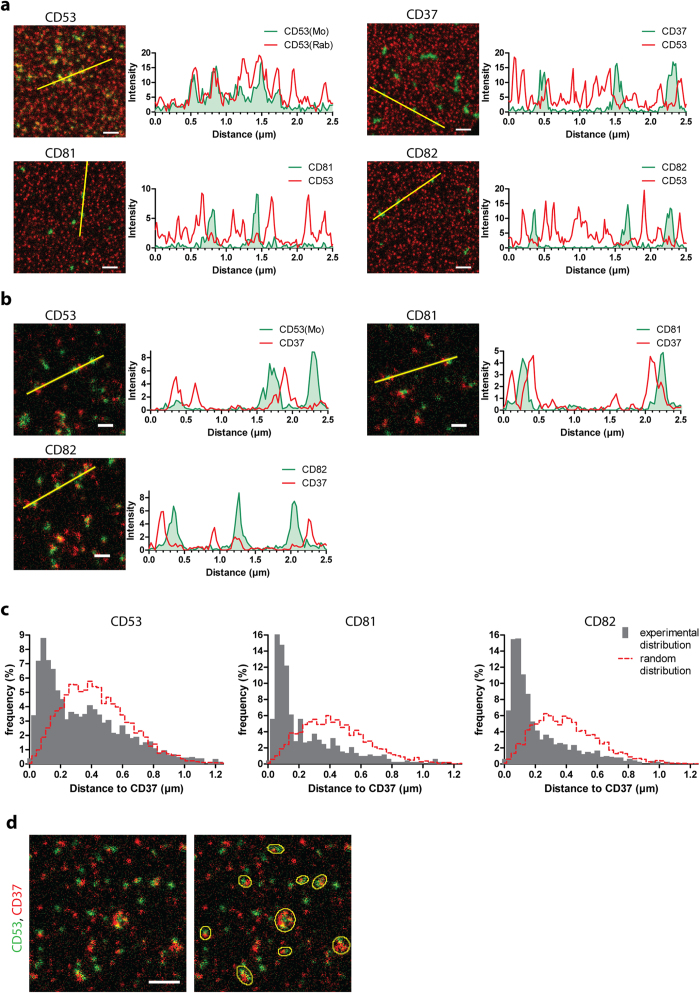
Tetraspanin clusters are organized at a higher spatial scale. **A** and **B**: Images: zoom of JY B cell membrane sheets double stained for CD53(Rab) (red) and CD53(Mo), CD37, CD81 or CD82 (green) (**A**) or CD37 (red) and CD53, CD81 or CD82 (green) (**B**) imaged by STED microscopy. Scale bars represent 0.5 μm. Graphs: intensity profiles of CD53(Rab) or CD37 (red curves) and CD53(Mo), CD37, CD81 or CD82 (filled green) as depicted in the corresponding images. **C**: Distances from the center of annotated clusters to the center of their nearest CD37 clusters were determined. The nearest neighbor curves were compared to distance curves (red dashed curves) determined from mock images generated with the corresponding cluster densities and sizes, but with completely random and uncorrelated cluster distributions. Clusters of at least 10 sheets were analyzed per condition. **D**: Zoom of a B cell membrane sheet double stained for CD53 and CD37. Yellow ellipses indicate CD53 and CD37 clusters that are in close proximity to each other. Scale bar represents 1 μm.

To further investigate the architecture of the tetraspanin web, we analyzed the distances between different tetraspanin clusters in more detail. Notably, clusters containing tetraspanin CD53 were not overlapping with CD37, CD81 or CD82 clusters, indicating the presence of distinct 120 nm sized clusters that contain only this single member of the tetraspanin family (Fig. 4a). Such clusters were also found when B cells were stimulated implying that this distribution is independent of cell activation (data not shown). Although CD37 domains overlapped partly with CD81 or CD82, most CD37 domains were isolated and not enriched in other tetraspanins (Fig. 4b). We investigated the positioning of CD53, CD81 and CD82 clusters to the CD37 clusters. For this analysis, we generated 2-color images in which we completely randomly positioned clusters with the densities and size distributions of CD37, CD53, CD81 or CD82 clusters (Fig. S6a). We then performed the same nearest neighbor analyses on these random and uncorrelated mock images as with the experimental STED micrographs and compared the distance distributions (Fig. 4c). We found that a large population of CD53, CD81 or CD82 clusters had much shorter distances to CD37 clusters than would be expected if the domains were randomly distributed relative to each other. This indicates that tetraspanin clusters are organized at a larger scale and that although clusters of different individual tetraspanin family members are not or only slightly overlapping, they are often located in close proximity to each other (Fig. 4d).

### Tetraspanin-partner interactions resolved by super-resolution microscopy

**Figure 5:**
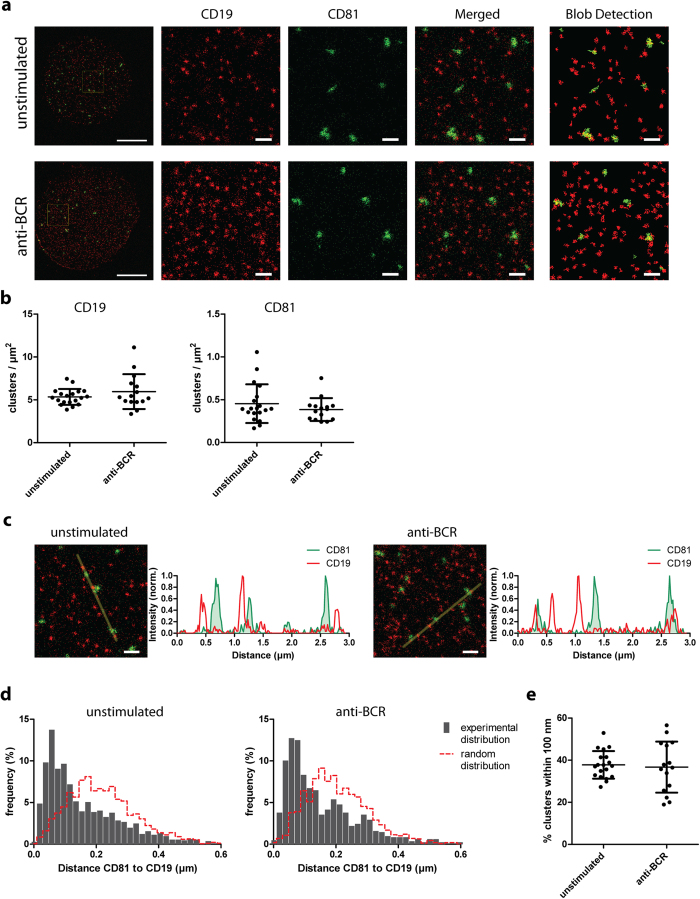
Organization of CD19 relative to CD81 on the plasma membrane of B cells. **A**: JY B cells were untreated or stimulated with anti-BCR for 5 min. Membrane sheets double stained for CD19 (red) and CD81 (green) were imaged by STED microscopy. Blob detection images (most right) show annotated clusters used for further analysis. Scale bars: 5 μm in whole sheets and 0.5 μm in zoomed and blob detection images. **B**: Surface density of the clusters from panel **A**. ≥15 sheets were analyzed per condition. Significance was tested with a Student T-test. **C**: Images: zoom of B cell membrane sheets double stained for CD19 (red) and CD81 (green), imaged by STED microscopy. Left: untreated B cells. Right: BCR-stimulated B cells. Scale bar: 0.5 μm. Graphs: intensity profiles of CD19 (red line) and CD81 (filled green) as depicted in the corresponding images. **D**: Distances from the center of annotated CD81 clusters to the center of their nearest CD19 cluster were determined. The nearest neighbor distance distributions were compared to distributions generated from 10 mock images with similar cluster densities and sizes but with completely random and uncorrelated cluster distributions. Clusters of ≥15 sheets were analyzed per condition. E: Percentage of CD19 clusters of which the distance from their center to the center of the nearest CD81 cluster was within 100 nm. Per sheet the percentage of clusters overlapping CD81 clusters was plotted for unstimulated and BCR-stimulated cells. Clusters of ≥15 sheets were analyzed per condition.

Since the interaction between CD19 and CD81 is crucial for efficient B cell activation via the B cell receptor (BCR), we investigated the nanoscale distribution of CD19 and CD81 clusters on the plasma membrane of B cells under resting conditions and upon BCR activation (Fig. 5a). The organization of CD81 and CD19 clusters relative to each other did not seem to change upon BCR crosslinking. In addition, the density of clusters remained unchanged upon B cell stimulation (Fig. 5b), and CD81 and CD19 did not become more co-localized into single domains (Fig. 5c). Still, CD81 clusters were in closer proximity to CD19 clusters than would be expected if the distribution of these clusters was random (Fig. 5d). Notably, we observed CD81 clusters to be in closer proximity to clusters of its interaction partner CD19 than to clusters of tetraspanin CD53 (Figs 5e and 3c).

**Figure 6:**
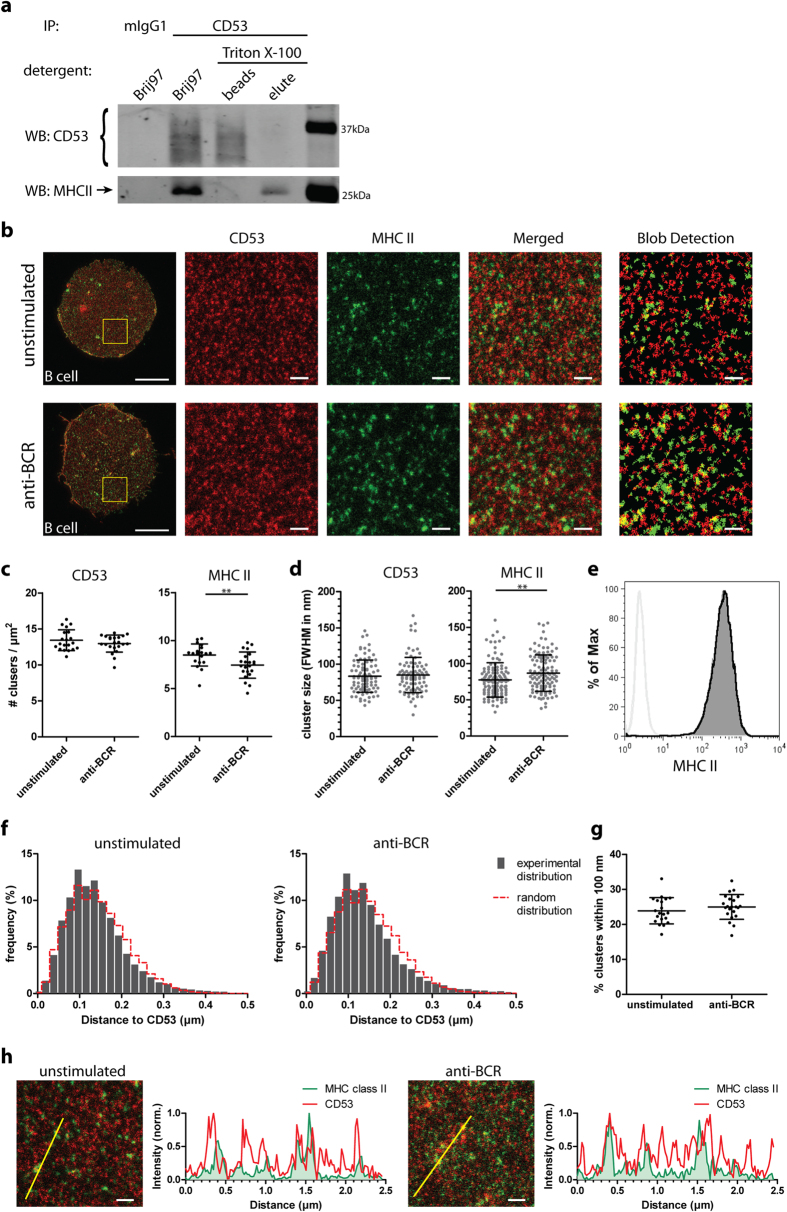
Organization of MHC class II relative to CD53 on the plasma membrane of B cells. **A**: CD53 was immunoprecipitated from JY B cell lysates as described in the Methods. The different fractions were investigated for the presence of CD53 (multiple bands as CD53 is glycosylated) and MHC II (arrow) by immunoblotting. **B**: B cells were untreated or stimulated with anti-BCR for 8 hours. Membrane sheets double stained for CD53 (red) and MHC II (green) were imaged by STED microscopy. Blob detection images (most right) show annotated clusters used for further analysis. Scale bars: 5 μm (whole sheets) and 0.5 μm (zoomed and blob detection images). **C**: Surface density of the clusters from panel **B**. ≥20 sheets were analyzed per condition. Significance was tested with a Mann Whitney test, p = 0.0079. **D**: The full-width at half-maximal intensity (FWHM) of CD53 and MHC II clusters. ≥120 random clusters were measured derived from 3 sheets per condition. Significance was tested with a Mann Whitney test, p = 0.0021. **E**: Unstimulated (filled gray) and stimulated B cells (black curve) were stained with MHC II or isotype control antibodies (light gray curves). Plasma membrane expression was analyzed by flow cytometry. **F**: Distances from the center of annotated MHC II clusters to the center of their nearest CD53 cluster were determined. The nearest neighbor distance distributions were compared to distributions generated from 10 mock images with similar cluster densities and sizes but with completely random and uncorrelated cluster distributions. **G**: Percentage of MHC clusters of which the distance from their center to the center of the nearest CD53 cluster was within 100 nm. Per sheet the percentage of clusters overlapping CD53 clusters was plotted for unstimulated and BCR-stimulated cells. F+G: Clusters of ≥20 sheets were analyzed per condition. **H**: Images: zoom of membrane sheets double stained for CD53 (red) and MHC II (green), imaged by STED microscopy. Scale bar: 0.5 μm. Graphs: intensity profiles of CD53 (red line) and MHC II (filled green) as depicted in the corresponding images.

Next, we investigated the localization of tetraspanin CD53 on antigen-presenting cells in relation to another well-known interaction partner of tetraspanins, namely MHC class II^7^,^8^,^33^,^34^. In accordance with the reported interaction between CD53 and MHC class II we were able to pull down endogenous MHC class II in protein complexes precipitated from lysates of B cells using antibodies against CD53 (Fig. 6a) in conditions that preserve tetraspanin-tetraspanin interactions (Brij97). This interaction was lost under conditions in which a strong detergent (Triton X-100) was used, often interpreted as an indirect interaction in the tetraspanin field. To investigate the nanoscale organization of endogenous MHC class II molecules, we visualized CD53 and MHC class II clusters on the plasma membrane of B cells with dual color STED microscopy (Fig. 6b). Upon activation of B cell via the BCR, the density and sizes of CD53 clusters remained unchanged. The density of MHC however decreased, while the determined average size of MHC clusters increased from 77 to 87 nm (Fig. 6c,d). This is likely due to coalescence of smaller clusters of MHC class II into larger clusters, since flow cytometry showed that the expression level of MHC on the plasma membrane of B cells did not change upon B cell activation (Fig. 6e). We investigated the proximity of MHC class II to CD53 clusters by comparing nearest neighbor distances of MHC class II to CD53, with distances calculated from computer generated mock images with a random distribution of both MHC class II and CD53 clusters (Fig. S6b). Both before and after B cell stimulation, MHC class II clusters were in slightly closer proximity to CD53 clusters than predicted from completely randomly positioned clusters (Fig. 6f,g). Moreover, a higher percentage of CD53 clusters was overlapping with MHC class II clusters than with clusters enriched in CD37, CD81 or CD82 (Figs 3c,4a,6g,h). These findings indicate that CD53 is in closer proximity to its interaction protein MHC class II than to other members of the tetraspanin family.

**Figure 7:**
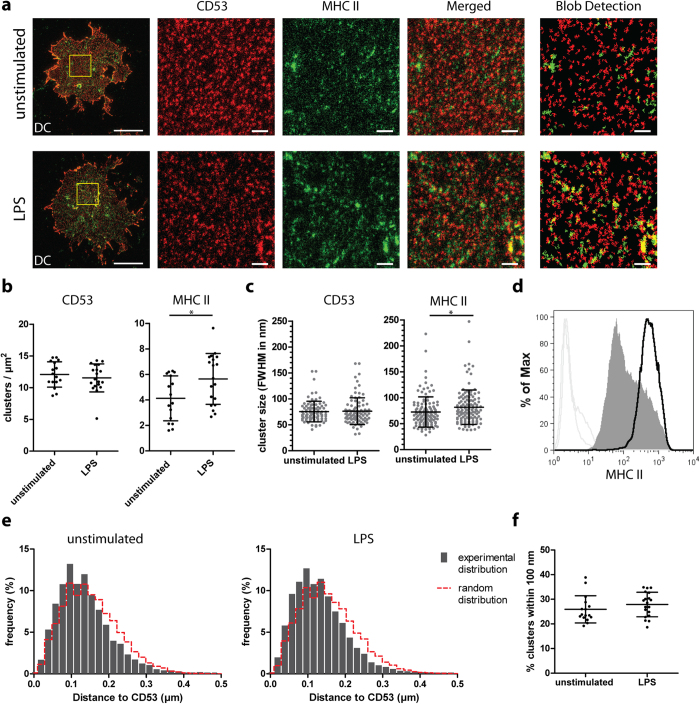
Organization of MHC class II relative to CD53 on the plasma membrane of DCs. Monocyte-derived DCs were untreated or stimulated with LPS for 24 hours. **A**: DC membrane sheets double stained for CD53 (red) and MHC class II (green) were imaged by STED microscopy. Middle images: magnification of the area indicated in whole sheets images (most left). Blob detection images (most right) show annotated clusters used for further analysis. Scale bars represent 5 μm in whole sheet images, and 0.5 μm in zoomed and blob detection images. **B**: Density of CD53 and MHC class II clusters from panel A. At least 16 sheets were analyzed per condition. Significance was tested with a Student T-test, p = 0.0236. **C**: The full-width at half-maximal intensity (FWHM) of CD53 and MHC II clusters. At least 120 random clusters were measured derived from 3 sheets per condition. Significance was tested with a Mann Whitney test, p = 0.0254. **D**: Unstimulated (filled gray) and stimulated monocyte-derived DC (black curve) were stained with MHC II or isotype control antibodies (light gray curves). Plasma membrane expression was analyzed by flow cytometry. **E**: Distances from the centers of annotated MHC class II clusters to the centers of their nearest CD53 cluster. The nearest neighbor distance distributions were compared to distributions generated from 10 mock images with similar cluster densities and sizes but with completely random and uncorrelated cluster distributions (red dashed curves). Clusters of at least 16 sheets were analyzed per condition. **F**: Percentage of MHC clusters of which the distance from their center to the center of the nearest CD53 cluster was less than 100 nm. Clusters of at least 16 sheets were analyzed for both unstimulated and LPS stimulated DCs. Significance was tested with a Student T-test.

To further address the nanoscale distribution of MHC class II relative to CD53 in primary cells, we studied human monocyte-derived dendritic cells (DCs) under resting and stimulatory conditions (Fig. 7a; stimulation with LPS). In line with B cells, clusters of individual members of the tetraspanin family, including CD9, did not co-localize with each other (Fig. S7a and b). Moreover the density and sizes of CD53 clusters on the plasma membrane of DCs remained unchanged upon activation with LPS (Fig. 7b,c). However, we observed that in DCs not only the size but also the density of MHC class II clusters increased upon cell activation (Fig. 7b,c). These data corroborate the finding that in DCs the plasma membrane expression of MHC class II was increased upon activation (Fig. 7d). To investigate the proximity of MHC class II clusters to CD53 in DCs, we generated mock images with random and uncorrelated cluster organizations based on the cluster sizes and densities obtained from the STED images (Fig. S6c). In line with the data obtained in B cells, MHC class II clusters were in closer proximity to CD53 compared to random distributions in both resting and activated DCs (Fig. 7e), and this proximity of MHC class II clusters to CD53 clusters did not significantly alter upon cell activation (Fig. 7f).

### Quantification of the number of tetraspanin molecules per cluster

**Figure 8:**
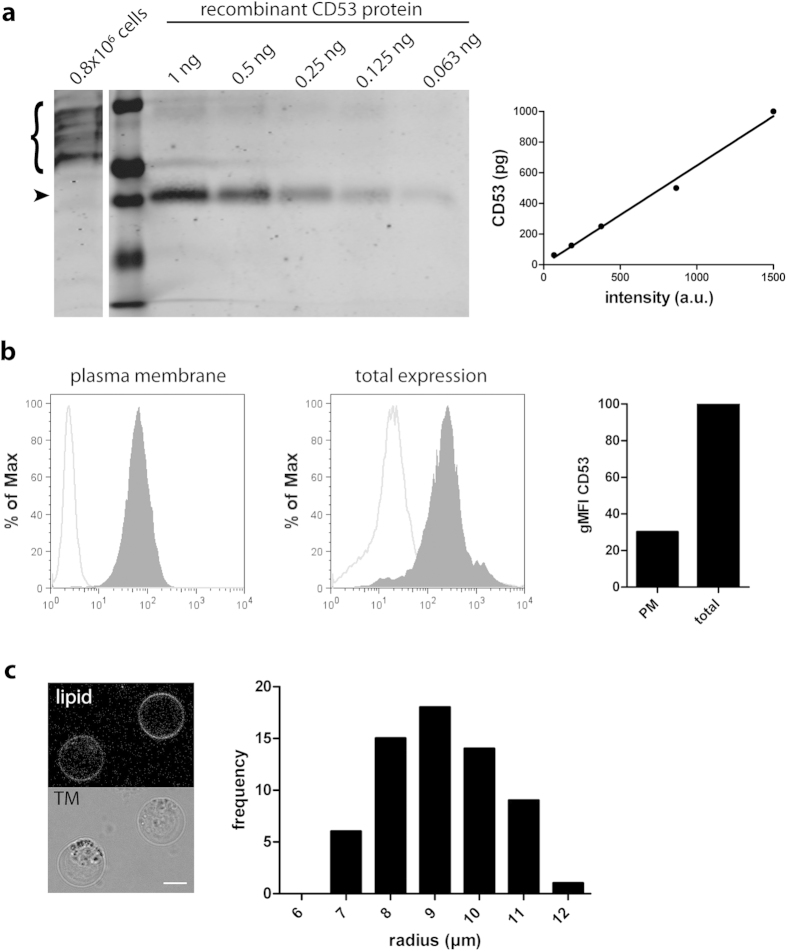
Quantification of the number of CD53 molecules per cluster. **A**: Quantitative Western blot to determine the number of CD53 molecules per cell. B cell lysate and titrated samples of recombinant CD53 protein were subjected to SDS-page and immunoblotted with CD53(Rab) antibody against the C-terminus of CD53. The difference in protein mass of cell-derived CD53 (curly bracket; multiple bands, 25–37 kDa) versus recombinant CD53 protein (22 kDa; arrowhead) is due to glycosylation. Graph: standard curve of CD53 recombinant protein determined by densitometric analysis. **B**: Expression of CD53 on the plasma membrane and on intracellular membranes. B cells were stained with CD53(Mo) to measure plasma membrane (left) and total CD53 expression (right). CD53 expression was determined by flow cytometry (filled gray histograms) and corrected for a-specific antibody binding (isotype control antibodies, light grey curves). Graph: expression of CD53 on the plasma membrane and in the total B cell, corrected for a-specific staining and normalized to total CD53 expression. **C**: Determination of the total surface area of B cells. Cell ruffles were eliminated by treating cells with Latrunculin A (5 μM) for 15 min and stretching the plasma membrane by decreasing the osmolarity of the imaging medium from 280 to 200 mOsm. Plasma membranes were stained with the lipophilic membrane dye DiI, and the diameter of spherical cells was measured at the equatorial plane by confocal microscopy. Scale bar represents 10 μm. Graph: frequency distribution of the radius of B cells, 63 cells were measured.

By investigating the tetraspanin web with dual color STED microscopy, we observed that the studied tetraspanin proteins were almost exclusively clustered with tetraspanins of the same species and these clusters were largely devoid of other members of the tetraspanin family. To investigate the biophysical nature of these tetraspanin clusters further, we estimated the number of CD53 molecules per CD53 cluster (Table 1). We first calculated the total amount of endogenous CD53 protein in a single B cell by quantitative immunoblotting using recombinant CD53 protein (Fig. 8a). On average, a total of 53,000 molecules of CD53 were present per B cell (Table 1). By immunolabeling B cells with the CD53-antibody recognizing the extracellular domain with or without membrane permeabilization, we estimated that 30% of these CD53 molecules localized to the plasma membrane whereas the remainder was present in intracellular membranes (Fig. 8b). After correcting for this intracellular pool of CD53 protein, we quantified that about 16,000 molecules of CD53 were expressed at the cell surface of a single B cell. To obtain the total number of clusters on the plasma membrane, we first estimated the average total cell surface area. To eliminate membrane inconsistencies, plasma membranes were stretched by disrupting the cytoskeleton with Latrunculin A and placing cells in hypotonic medium (Fig. 8c). The radius at the equatorial plane of these spherical cells was measured to determine an average cell surface area of 1,040 μm^2^. Since STED microscopy analysis revealed that the CD53 cluster density was 4.4 clusters / μm^2^ in the plasma membrane (Fig. 2d), this indicates that a single B cell contains on average approximately 4,500 CD53 clusters per cell. Consequently, we estimated that each of these clusters contains on average 3.5 molecules of CD53 on the cell surface of B cells. Although this value should be taken with great caution due to cellular heterogeneities and an experimental error in any of these steps cannot be excluded, we believe that it is warranted to conclude that the number of CD53 molecules per domain is lower than 10.

## Discussion

The superfamily of tetraspanin proteins is important in many fundamental cell biological processes, still the physical principles of the tetraspanin network in the plasma membrane are not adequately understood. The current hypothesis is that tetraspanins assemble multi-molecular complexes (TEMs) composed of different tetraspanins and their interacting partner molecules that together build ‘the tetraspanin web’. Tetraspanins can interact with other tetraspanins in the plasma membrane as evidenced by biochemical and microscopy-based studies in many different cell types, including immune cells (reviewed in^6^). These tetraspanin-tetraspanin interactions have been reported to be regulated by lipids, including gangliosides and cholesterol^35^,^36^, and by palmitoylation of intracellular cysteine residues^31^,^37^,^38^ that may also modulate tetraspanin dynamics^19^ and their cluster size^23^. Recently, the δ-domain of the large extracellular loop of tetraspanin CD81 was shown to be important for CD81 cluster formation^39^. In several studies it was estimated that multiple (>3) different members of the tetraspanin family could exist in one single complex^3^,^7^,^8^,^40^. Still the organization of multiple members of the tetraspanin family in native plasma membranes has not been studied at the nanoscale level.

**Figure 9:**
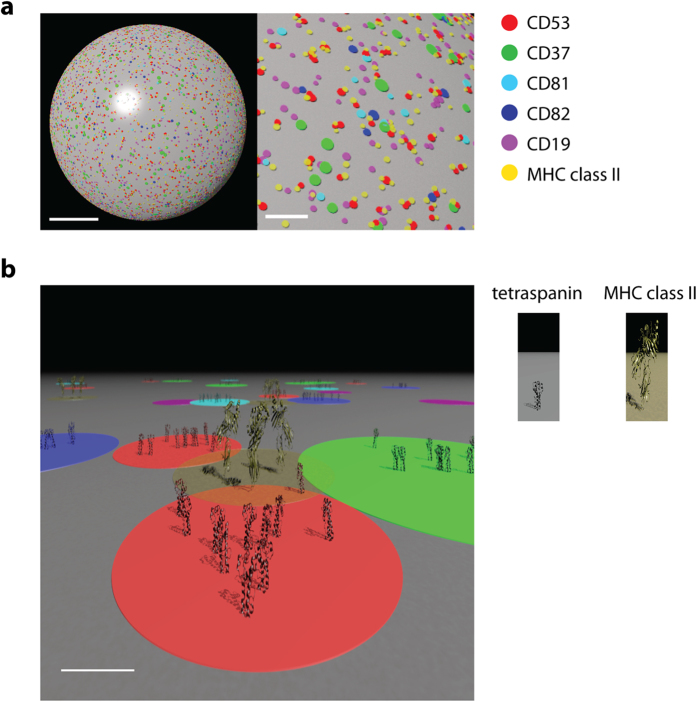
The tetraspanin web revisited. **A**: Model of the spatial organization of tetraspanin clusters on the plasma membrane of a B cell. Based on the cluster sizes, densities and distributions derived from the STED images, CD53, CD37, CD81, CD82, CD19 and MHC class II clusters were modeled on the surface of a B cell. Scale bars represent 5 μm (left) and 1 μm (right). **B**: Model of the nanoscale organization of TEMs on the plasma membrane. Separate clusters enriched in either CD53 (red), CD37 (green), CD81 (cyan) or CD82 (blue). Each cluster contains below 10 tetraspanin molecules of a single tetraspanin species. CD53 clusters are excluded from clusters containing CD37, CD81 or CD82. In contrast, clusters of MHC class II molecules (yellow, transparent) are partly overlapping with tetraspanin clusters, facilitating regulation of the function of MHC class II by multiple members of the tetraspanin family. Similarly, CD19 (purple) clusters overlap with CD81 clusters. Clusters of different tetraspanin species are adjacently positioned and can dynamically interact with each other forming a functional tetraspanin web in the plasma membrane. Scale bar represents 20 nm.

To resolve the architecture of the endogenous tetraspanin web at the cell surface, we employed dual color STED microscopy. Here, we elucidated the size, distribution and stoichiometry of tetraspanin clusters in human immune cells and propose a new view on the tetraspanin web (Fig. 9). Based on the densities, distribution and size of clusters containing four different members of the tetraspanin family and the interaction partners CD19 and MHC class II, we modeled the spatial organization of the tetraspanin domains on the plasma membrane of a human B cell (Fig. 9a). Based on our findings, tetraspanin clusters are non-randomly distributed on the plasma membrane. In contrast to the current dogma of a TEM, we observed that individual members of the tetraspanin family (CD37, CD81 and CD82) are present in nanoscale clusters that are largely devoid of other members of the tetraspanin family (CD53). The clusters detected in this study had an average size of below 120 nm and contained less than 10 molecules of one member of the tetraspanin family (Fig. 9b). This size is comparable to the TEM size (CD63 in HeLa cells; 190 nm) reported in former electron microscopy studies^40^ and recent STORM studies (CD82 in KG1a cells; 90 nm^23^). Our findings are in line with the tendency of tetraspanin proteins to undergo homotypic interactions. Covalently crosslinking tetraspanins CD9, CD81 and CD151 showed that the level of homodimers exceeded the level of crosslinking between different tetraspanin members (heterodimers), and CD9 homodimers were found to assemble in the Golgi and subsequently expressed at the cell surface^41^. Studies using detergents of different strength showed that heterotypic tetraspanin-tetraspanin interactions are relatively weak^42^,^43^. Moreover, members of the tetraspanin family can have different localizations within the cell membrane as shown by CD9 localization that has an opposite polarity to CD151 in cells of the stratum basale in skin^44^.

We now show for the first time in native plasma membranes that different members of the tetraspanin superfamily are indeed not intimately interacting with CD53 or CD37 in membrane clusters. Since it is estimated that leukocytes express 20 different tetraspanins^45^, we do not exclude that the distribution of tetraspanin proteins not studied here may be different, as also indicated by our finding that CD37 domains show more (but still little) overlap with CD81 and CD82 compared to CD53. Based on the four different tetraspanins studied here, we propose that tetraspanin homo-oligomers together with their primary non-tetraspanin interaction partner (for example CD81-CD19) form the basic building blocks for a stable tetraspanin cluster (e.g. TEM). These clusters form a dynamic network across the plasma membrane involving weak heterotypic interactions with other tetraspanin clusters or other non-tetraspanin interaction partners (the tetraspanin web), resulting in a non-random distribution of clusters of different tetraspanin members adjacent to each other. Such a model is supported by elegant single molecule tracking studies showing that tetraspanin interactions are highly dynamic and distinct diffusion modes of tetraspanins were observed which could correspond to different clustering states^19^.

Tetraspanin proteins can control the function of their interacting partners by influencing the clustering of partner proteins^21^,^23^. In our study we found MHC class II molecules located in clusters on the plasma membrane of both B cells and primary DCs. These clusters likely enable the local high avidity of MHC class II that is crucial for efficiently activating T cells. Despite the finding that the interaction of MHC class II with CD53 on B cells is relatively weak (e.g. not preserved in harsh detergent conditions), MHC class II clusters were non-randomly distributed in close proximity to CD53 clusters. We observed MHC-II cluster size to increase upon DC and B cell activation, in contrast to the cluster size of CD53 which was unaltered. This suggests that CD53 plays no role in activation-induced MHC-II clustering, although we cannot exclude the role of other tetraspanin members in this process. We envision secondary tetraspanin partners to be localized between the stable primary tetraspanin clusters, where their function can be regulated by multiple members of the tetraspanin family in a dynamic manner (Fig. 9).

Taken together, we visualized the tetraspanin network including interacting partner proteins on the cell surface of immune cells with super-resolution microscopy. Since the molecular basis of membrane segregation into microdomains in the plasma membrane is not well understood, dual color super-resolution microscopy techniques may also be applied to study other protein families that are postulated to cluster together into protein islands. A detailed molecular understanding of TEMs at the nanoscale level will significantly contribute to better comprehension of the molecular mechanisms underlying tetraspanin biology in immune cells and other cell types.

## Materials and Methods

### Antibodies

Two antibodies were used for the detection of CD53: a monoclonal rabbit antibody against the C-terminus of human CD53 (EPR4342(2), LifeSpan BioSciences, referred to as CD53(Rab)) and a mouse antibody against the extracellular loop of human CD53 (referred to as CD53(Mo), both unlabeled (mem53, Serotec) and FITC-labeled (mem53-FITC, Abcam)). Mouse primary antibodies against human CD9 (M-L13, BD Biosciences), CD37 (WR-17, in house produced), CD81 (JS-81, BD Biosciences), CD82 (B-L2, Serotec), CD82(Rab) (AHP1709, Serotec), CD55 (143-30, eBioscience), CD19 (#3574, Cell Signaling Technology) and MHC class II (Q5/13, in house produced) were used. Isotype control antibodies were mouse IgG1(X40, BD Biosciences) and rabbit IgG (Jackson ImmunoResearch). Alexa488 or Alexa647 goat anti-mouse IgG (Invitrogen) was used for detection by flow cytometry. Goat anti-rabbit and anti-mouse F(ab) fragments (111-007-003 and 115-007-003, Echelon) were labeled by incubating 200 μl of 50 μM F(ab) fragment with a 50-fold molar excess of Atto594 NHS-ester (Attotec). Unconjugated dye molecules were removed by size exclusion chromatography with sephadex G50 to 10 mM Na-phosphate buffer with 250 mM NaCl at pH 7.6. Goat anti-rabbit, sheep anti-mouse and goat anti-mouse antibodies (Dianova) were labeled with Atto594 or KK114 (Abberior Red, Abberior). For experiments in Fig. 3b, goat anti-mouse IgG1-Atto590 (Enzo Life Sciences) was used, and rat anti-mouse IgG2a (R19-15, BD Biosciences) was labeled by incubating 70 μl of 0.5 mg/ml antibody with a 40-fold molar excess of KK114-NHS-ester (Abberior) at pH 8. Unconjugated dye molecules were removed on 40K MWCO Zeba Spin desalting columns (Thermo Scientific) at pH 7. Cortical actin was stained with phalloidin-Alexa546 (Invitrogen).

### Cell culture and preparation of human DCs

The human B cell line JY was maintained in RMPI1640 medium with 10% Fetal Bovine Serum (Greiner Bio One), stable Glutamine (PAA) and Antibiotic-Antimycotic (Gibco). Where indicated, JY cells were stimulated with 1 μg/ml anti-BCR antibody for 8 hours, 10 μg/ml for 5 min, or treated with 5 μM Latrunculin A (LatA) for 15 min at 37 °C. DCs were generated from peripheral blood mononuclear cells as described previously^46^. Plastic-adherent monocytes were cultured in RMPI1640 medium with 10% Fetal Bovine Serum, stable Glutamine and Antibiotic-Antimycotic, IL-4 (300 U/ml) and GM-CSF (450 U/ml) for 7 days. At 6 days of culture, immature DCs were stimulated with 100 ng/ml LPS for 24 hours.

### Membrane sheet production and antibody staining

For the preparation of the membrane sheets, JY B cells were kept at 4 °C until membrane fixation to prevent antibody induced clustering of membrane proteins. Stimulated or unstimulated cells were washed with cold PBS and blocked in 3% BSA, 1% filtered human serum and 10 mM glycine in PBS. Extracellular epitopes on cells were stained with mouse primary antibodies, followed by staining with anti-mouse secondary antibodies in blocking solution at 4 °C. Coverslips were cleaned in 1% Hellmanex III (Hellma) and sonified for 15 min at RT, rinsed with ultrapure water, blow dried, and coated with poly-L-lysine. Thoroughly washed cells were seeded on the coverslips for 15 min at 4 °C. Non-adhered cells were removed by a gentle wash step with cold PBS. Coverslips were covered with cold PBS and a sonic pulse of 0.1 s at 10% power was applied (sonifier, Branson). Cell debris was washed away with cold PBS and membrane sheets were immediately fixed in 2% PFA in PBS. Sheets were washed with PBS and blocked, and stained with CD53(Rab), CD19 or CD82(Rab) (intracellular epitopes) followed by staining with anti-rabbit secondary antibodies. Coverslips were washed and embedded in Mowiol (Sigma). As the mouse antibodies used in this study recognize extracellular domains of membrane proteins and the rabbit antibodies bind intracellular domains, we can rule out binding artifacts due to steric hindrance. Note that no detergent was used for the preparation of the membrane sheets.

### Whole cell immunostaining

Raji B cells were adhered to poly-L-lysine coated coverslips and fixed in 2% PFA. Cells were blocked in 3% BSA, 1% filtered human serum and 10 mM glycine in PBS. Membranes were permeabilized with 0.5% saponin, 3% BSA, 1% filtered human serum and 10 mM glycine in PBS. Cells were stained with CD53(Mo) in saponin containing block buffer and washed, followed by staining with sheep-anti-mouse Ig-KK114. Coverslips were washed with saponin buffer, PBS and ultrapure water, and embedded in Mowiol.

### Dual color STED and confocal imaging

The dual color STED setup is described in detail in^26^. In short, excitation of the Atto594 and KK114 dyes was performed with two pulsed diode lasers at 595 nm and 640 nm (PicoQuant). STED was performed using a frequency-doubled fiber laser (ELP-5-775-DG, IPG Photonics Corporation) emitting at 775 nm at a repetition rate of 20 MHz. Excitation pulses were synchronized to the STED pulses. The excitation beams and the donut shaped STED beam were co-aligned and coupled into a 1.4 numerical aperture oil immersion lens (NA 1.4 HCX PL APO, 100x, Leica Microsystems). The fluorescence was collected by the same lens, spectrally separated and filtered into two ranges: 600–640 nm and 650–690 nm. The fluorescence was detected by fiber-coupled single-photon-counting modules (SPCMAQRH13, Perkin Elmer), with their fiber cores acting as confocal pinholes. Images with 20 nm sized pixel steps were acquired and the hardware and data acquisition was controlled by the software ImSpector (http://www.imspector.de).

### Image analysis

All image analysis was performed using Fiji software^47^. Edges of sheets were excluded from analysis. For determination of the cluster size, random clusters were selected from representative sheets and an intensity profile was plotted for each cluster. The width of the profile at half-maximal intensity was measured as cluster size. For area and circularity measurements, regions of interest were created corresponding to tetraspanin clusters by applying a threshold to STED images of membrane sheets. Of these regions the area and circularity was determined. To annotate the location of tetraspanin clusters on a sheet, a simple intensity threshold was applied to the STED images followed by a blob detection algorithm. The x/y positions of the centers of the tetraspanin clusters were then used to determine the distances to the nearest neighboring clusters. These nearest neighbor distances to tetraspanin clusters of the same species were fitted with random distance distribution curves (Originlab). Assuming uncorrelated cluster distributions, the probability distribution d*p* for finding the nearest neighbor at a distance *r *± d*r* is given by:


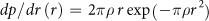


For the fitting, the density *ρ* was independently determined from the images (i.e. by dividing the number of domains over the area of analyzed plasma membrane). To obtain nearest neighbor distance distributions for two different tetraspanin species (or from CD53 to CD55 or MHC class II), mock images were generated by a custom program in VB.NET. In these mock images, domains were randomly placed with the densities and size distributions determined from the STED images. For obtaining the nearest neighbor histogram, these mock images were then analyzed identical to the STED images as described above.

### Immunoprecipitation

JY B cells were lysed in lysisbuffer containing 1% detergent (Brij97), 10 mM Tris (pH 7.5), 150 mM NaCl, 2 mM MgCl_2_, 2 mM CaCl_2_, 5 mM NaF, 1 mM Na_3_VO_4_ with protease inhibitors (EDTA free, Roche). Cell lysates were precleared with bare protein G sepharose beads (GE Healthcare) and isotype control antibody-bound beads. Immunoprecipitation was performed with CD53(Mo)-bound beads and isotype control antibody-bound beads. After incubating with the lysate for 2 hours at 4 °C, beads were washed in lysis buffer. A fraction of the beads was incubated with 1% Triton X-100 to elute weak interaction partners from CD53 immunocomplexes, and eluted proteins were acetone precipitated. Samples were boiled at 95 °C, separated by SDS-PAGE under non-reducing conditions and blotted on polyvinylidene difluoride (PVDF) membranes (Millipore). Membranes were blocked with 3% BSA and 1% skim milk powder in TBS and probed with CD53(Mo) (mem53) and MHC class II (Q5/13) antibodies overnight at 4 °C. Detection was performed with IRDye-conjugated secondary antibodies and the Odyssey infrared detection system (LI-COR).

### Flow cytometry

Stimulated and unstimulated DC and JY B cells were stained with primary antibodies against tetraspanin proteins or MHC class II or isotype control antibody and subsequently stained with Alexa488-labeled goat anti-mouse-IgG in PBS containing 1% BSA and 0.01% NaN_3_ (PBA) with 2% human serum. Geometric mean fluorescence intensity was measured using flow cytometry (FACS Calibur, BD Biosciences) and analyzed using FlowJo. To determine the plasma membrane fraction of CD53, JY cells were first stained with CD53(Mo) or isotype control antibody followed by staining with Alexa488 labeled goat-anti-mouse IgG in 2% human serum PBA at 4 °C. To determine total CD53 expression, cells were fixed in 2% PFA, permeabilized in 0.5% saponin PBA, and stained with CD53(Mo) or isotype control antibody and Alexa488-labeled goat anti-mouse-IgG in 2% human serum 0.5% saponin PBA at 4 °C.

### Quantitative Western Blotting

JY cells were lysed at 20 × 10^6^ cells/ml in Laemmli sample buffer containing 5% β-mercaptoethanol. Human CD53 recombinant protein (Abnova) was titrated in sample buffer. Samples were boiled at 95 °C, separated by SDS-PAGE and blotted on PVDF membranes. Membranes were blocked with 3% BSA and 1% skim milk powder in TBS and probed with CD53(Rab) antibodies overnight at 4 °C. This was followed by IRDye-conjugated secondary antibodies and Odyssey infrared detection.

### Confocal microscopy to determine surface membrane area

JY cells were seeded in Willco dishes (Willco Wells) in 281 mOsm PBS, and treated with 5 μM Latrunculin A for 15 min at 37 °C. Plasma membranes were stained with fluorescent lipophilic tracer DiI (1,1′-Dioctadecyl-3,3,3′,3′-Tetramethylindocarbocyanine Perchlorate (DiIC_18_(3), Molecular Probes) (100 μM, DiI was stored at 10 mM in EtOH). A solution of 10 mM NaPO_4_ was titrated to the cell suspension to obtain a hypotonic cell environment of 196 mOsm and to eliminate plasma membrane ruffles. Spherical cells were imaged on a SP8 confocal microscope equipped with a 60× water 1.2 NA objective (Leica, Wetzlar, Germany) using appropriate laser lines and settings. Osmolarities were determined by freezing point depression (Osmomat 3000, Gonotec).

### Modelling

Model images of the tetraspanin domains were generated with POVray. The predicted structure of CD81^48^ (protein database accession number 2AVZ) and the crystal structure of MHC class II^49^ (protein database accession number 4IS6) were used.

### Statistics

Statistical analysis was carried out with Graphpad Prism. Data are presented as mean ± standard deviation for column scatter plots and median ± interquartile range for box plots (with whiskers representing the smallest and largest value). Student T-tests were used to compare two groups and an one-way analysis of variance (ANOVA) with a post hoc Bonferroni’s multiple comparison test was executed to compare the means of more than two groups. Data that did not pass the Kolmogorov-Smirnov normality test were compared with a Mann Whitney test (for two groups) or a Kruskal-Wallis test followed by Dunn’s multiple comparison testing. Statistical significance was defined as *p ≤ 0.05, **p ≤ 0.01, and ***p ≤ 0.001.

## Additional Information

**How to cite this article**: Zuidscherwoude, M. *et al.* The tetraspanin web revisited by super-resolution microscopy. *Sci. Rep.* **5**, 12201; doi: 10.1038/srep12201 (2015).

## Supplementary Material

Supplementary Information

## Figures and Tables

**Table 1:** Approximation of the number of CD53 molecules per cluster.

Amount of total CD53 in 0.8 × 10^6^ cells	Mass of CD53 protein	CD53 proteins/ cell	Fraction of total CD53 on plasma membrane	Surface area of the plasma membrane	Density of CD53(Mo) clusters
1,638 pg	23.34 kDa 4,042 × 10^−8^ pg	53,185	30.12%	1,040 μm^2^	4.4 clusters/μm^2^
