# Supplementary material for: The tetraspanin web revisited by super-resolution microscopy
Source: Sci Rep. 2015 Jul 17;5:12201. doi: 10.1038/srep12201 (PMC4505338; doi:10.1038/srep12201)
Supplement: Supplementary Information [file srep12201-s1.pdf]

Supplementary information

The tetraspanin web revisited by super-resolution microscopy

Malou Zuidscherwoude, Fabian Göttfert, Vera Marie E. Dunlock, Carl G. Figdor, Geert van den Bogaart, Annemiek B. van Spriel

Supplementary Figure legends

Figure S1: Whole cell STED imaging and antibody specificity.

A: CD53 visualized by STED microscopy on an intact B cell (left) and a B cell derived membrane sheet (right). Left: Raji B cells were permeabilized and stained with CD53(Mo) which was detected by anti-mouse KK114. Right: membrane sheet stained with CD53(Mo) which was detected by anti-mouse Atto594. Both images were acquired with 10 nm sized pixel steps. Scale bars represent 2 µm.

B: Membrane sheets were stained with phalloidin, fixed, and imaged by epifluorescent microscopy. Scale bar is 5 µm.

C: Images: B cells were treated with latrunculin A (LatA) or vehicle (DMSO) for 5 min. Membrane sheets double stained for CD53 (red) and CD37 (green) were imaged by STED microscopy. Scale bars: 5 µm in whole sheets and 0.5 µm in zoomed images. Graphs: Nearest neighbor analysis. Distance distributions of CD37(Mo) clusters of DMSO (blue) or LatA (pink) treated cells to the nearest CD53(Rab) clusters from at least 11 sheets. For every sheet the percentage of clusters within 100 nm from the nearest CD53(Rab) cluster was plotted.

D: B cell membrane sheets were stained for CD53(Rab) and mouse IgG1 control antibody (upper), or Rabbit IgG control antibody and CD53(Mo). Rabbit antibodies were detected with anti-rabbit KK114 secondary antibodies and mouse antibodies were detected with anti-mouse Atto594. Scale bars represent 5 µm (sheet overview, left) or 0.5 µm (zoomed images).

E: Membrane sheets of JY, HEK293 and LN229 cells were stained with CD53(Rab) or isotype control antibodies, and visualized by a lipid dye TMA-DPH. Sheets were imaged by epifluorescent microscopy. Scale bars represent 5 µm.

F: B cells were stained simultaneously with CD53(Mo) and CD53(Rab) antibodies and analyzed by FACS. The red dashed line depicts the expected trend when CD53(Mo) antibody would have had lower affinity for CD53 protein than the CD53(Rab) antibody. Instead, a linear correlation in staining intensities was observed.

Figure S2: Blob detection and secondary antibody dye swap.

A: B cell membrane sheets were stained for CD53(Rab) (upper) and CD53(Mo) (lower). Left: CD53 clusters visualized by STED microscopy. Right: clusters annotated by blob detection. Graph: intensity profile of CD53 on the line indicated in the images. Positions of the detected clusters are indicated in shaded areas. Most left, merged picture in which yellow indicates overlap. Scale bar represents 0.5 µm.

B: B cell membrane sheets were stained for CD53(Rab) and CD53(Mo) (upper), or CD53 (Rab) and CD55 (lower) and imaged with conventional confocal microscopy or by STED microscopy. Rabbit primary antibodies were visualized with anti-rabbit Atto594 secondary antibodies. Mouse primary antibodies were detected with anti-mouse KK114 secondary antibodies. Merged image of CD53(Rab) (green) and CD53(Mo) or CD55 (red) in which yellow indicates overlap. Right images show clusters that were annotated by blob detection. Graphs show distance distributions of CD53(Mo) (left) or CD55 (right) clusters to the nearest CD53(Rab) cluster. Green curves: sheets were stained with CD53(Mo) or CD55 which was detected by anti-mouse Atto594, and CD53(Rab) detected by anti-rabbit-KK114. Red curves: secondary antibody dyes were swapped. At least 10 sheets were analyzed per condition.

Figure S3: Secondary antibody controls.

A and B: B cell membrane sheets stained with CD53 primary antibodies (A: CD53(Rab); B: CD53(Mo)) and Atto594-labeled F(ab)-fragments (upper) or whole secondary antibodies (lower). Two representative sheets are shown for each condition. Graph: intensity profiles of CD53 clusters detected with F(ab) (upper, red) or whole antibody (lower, black) on the line depicted in images, to visualize cluster size. Scale bars represent 5 µm in sheets overview images and 0.5 µm in zoomed images.

C: Staining intensity of anti-mouse secondary antibody correlated with the staining intensity of CD53(Mo)-FITC. Left: FACS analysis of CD53 expression on whole B cells. Middle: B cells were stained with CD53(Mo)-FITC and anti-mouse-alexa647, followed by membrane sheets preparation. Right: intensity profile of FITC and alexa647 staining on the line depicted in the middle images.

D: B cells were labeled for CD37 or CD53 (single labeled cells), or for CD37 and CD53 simultaneously (dual labeled cells) and analyzed for staining intensity of anti-CD53 (mIgG1) and anti-CD37 (mIgG2a). B cells were stained with CD53(Mo, mIgG1) and/or CD37 antibodies (mIgG2a), followed by staining with anti-mIgG1-alexa647 and anti-mIgG2a-alexa488 (left) or anti-mIgG1-Atto594 and anti-mIgG2a-KK114 (right).

Figure S4: Expression of CD37, CD53, CD81 and CD82 on the plasma membrane of B cells.

Unpermeabilized cells were stained for tetraspanin proteins and expression was determined by flow cytometry (filled gray histogram) and corrected for a-specific antibody binding (isotype control antibodies, light grey line). Graph: expression of CD53, CD37, CD81 and CD82 on the plasma membrane of B cells, corrected for a-specific staining, mean fluorescent intensity is depicted.

Figure S5: CD53 does not overlap with CD82.

A: B cell membrane sheets double stained for CD82(Rab) (red) and CD82(Mo) (green) were imaged by STED microscopy. Scale bars: 5 µm in whole sheets and 1 µm in zoomed images. Graph: intensity profiles of CD82(Rab) (red curves) and CD82(Mo) (filled green) as depicted in the corresponding images.

B: B cells membrane sheets double stained for CD82(Rab) (red) and CD53(Mo) (green) were imaged by STED microscopy. Scale bars: 5 µm in whole sheets and 0.5 µm in zoomed images. Graphs: intensity profiles of CD82 (red line) and CD53 (filled green) as depicted in the corresponding images.

Figure S6: Mock images of random and uncorrelated cluster distributions.

A: Mock images with completely random and uncorrelated cluster distributions that were simulated using the size distributions and densities of tetraspanin clusters determined from the STED microscopy images of B cell membrane sheets. Distributions of CD53(Rab) clusters (red) with CD53(Mo), CD81 or CD82 clusters (green) were generated. Scale bars represent 5 µm in overview images and 0.5 µm in zoomed images.

B: Mock images with random cluster distributions that were generated using the size distributions and densities of CD53(Rab) and MHC class II clusters determined from the STED microscopy images of membrane sheets derived from untreated and BCR stimulated B cells. Distributions of CD53(Rab) clusters (red) and MHC class II clusters (green) were generated. Scale bars represent 5 µm in overview images and 0.5 µm in zoomed images.

C: Mock images with random cluster distributions that were generated using the sizes, size distributions and densities of CD53(Rab) and MHC class II clusters determined from the STED microscopy images of membrane sheets derived from untreated and LPS stimulated DCs. Distributions of CD53(Rab) clusters (red) and MHC class II clusters (green) were generated. Scale bars represent 5 µm in overview images and 0.5 µm in zoomed images.

Figure S7: CD53 and CD9 are not overlapping on the plasma membrane of DCs.

A: B cells were untreated or stimulated with LPS. Membrane sheets double stained for CD53(Rab) (red) and CD9 (green) were imaged by STED microscopy. Scale bars: 5 µm in whole sheets and 0.5 µm in zoomed and blob detection images.

B: Intensity profiles of CD53 (red line) and CD9 (filled green) as depicted in the corresponding images of A.

C: Percentage of CD9 clusters of which the distance from their center to the center of the nearest CD53 cluster was within 100 nm. The percentage of clusters overlapping CD53 clusters was plotted per sheet. Clusters of >6 sheets were analyzed per condition.

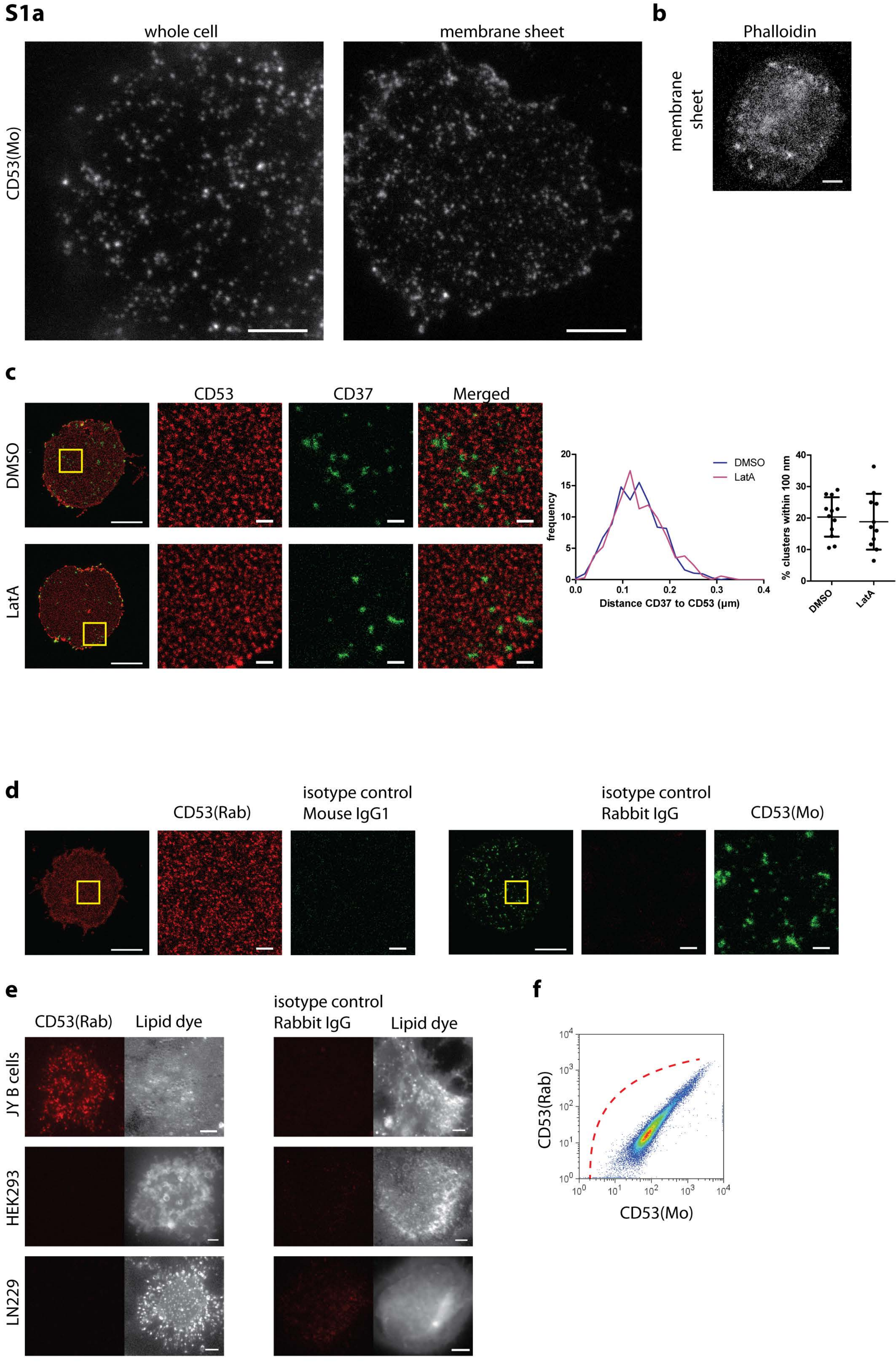

S1a
whole cell
membrane sheet
CD53(Mo)
b
Phalloidin
membrane sheet
c
CD53
CD37
Merged
DMSO
LatA
frequency
Distance CD37 to CD53 (µm)
% clusters within 100 nm
d
CD53(Rab)
isotype control Mouse IgG1
isotype control Rabbit IgG
CD53(Mo)
e
CD53(Rab)
Lipid dye
isotype control Rabbit IgG
JY B cells
HEK293
LN229
f
CD53(Rab)
CD53(Mo)

S2a

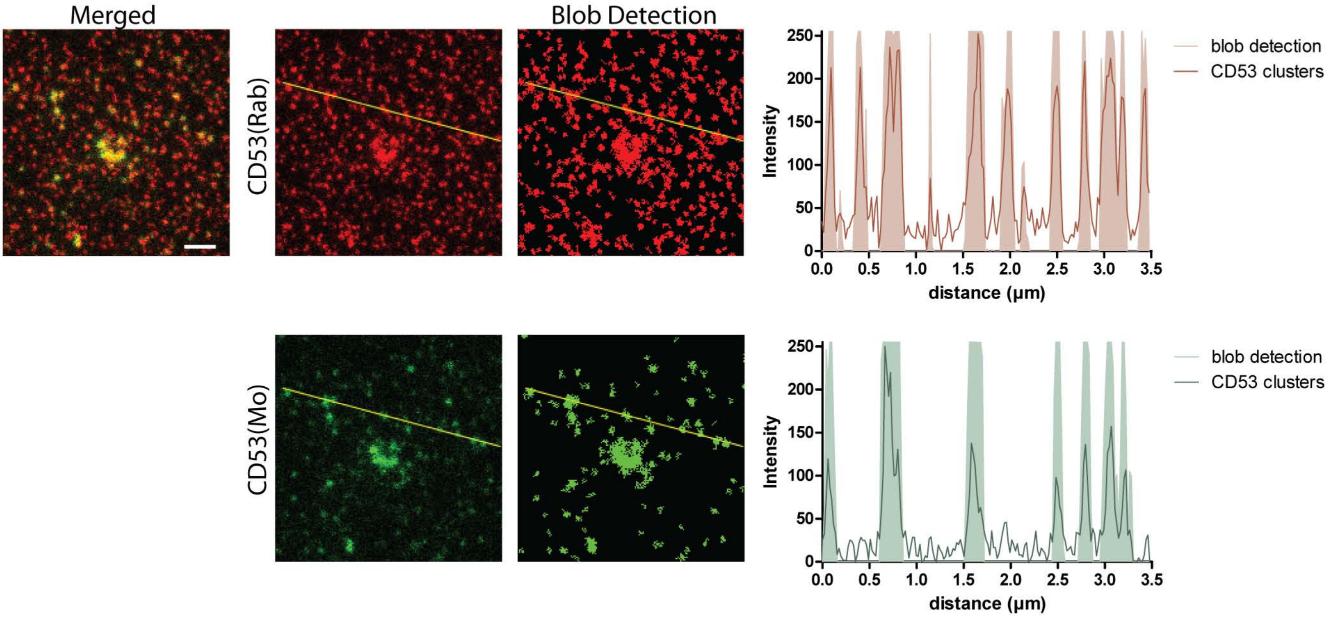
Merged
Blob Detection
CD53(Rab)
CD53(Mo)
blob detection
CD53 clusters
Intensity
distance (µm)
0.0
0.5
1.0
1.5
2.0
2.5
3.0
3.5
0
50
100
150
200
250

b

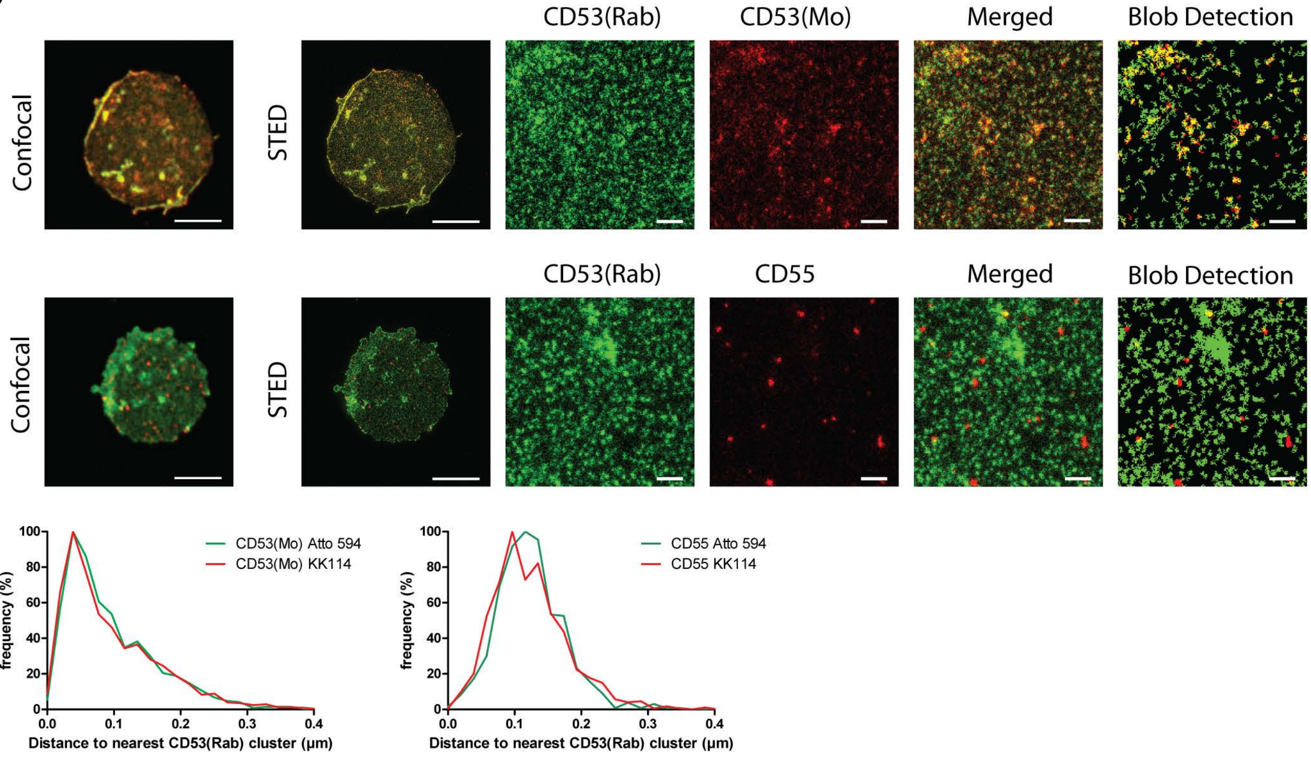
Confocal
STED
CD53(Rab)
CD53(Mo)
Merged
Blob Detection
CD55
CD53(Mo) Atto 594
CD53(Mo) KK114
CD55 Atto 594
CD55 KK114
frequency (%)
Distance to nearest CD53(Rab) cluster (µm)
0.0
0.1
0.2
0.3
0.4
0
20
40
60
80
100

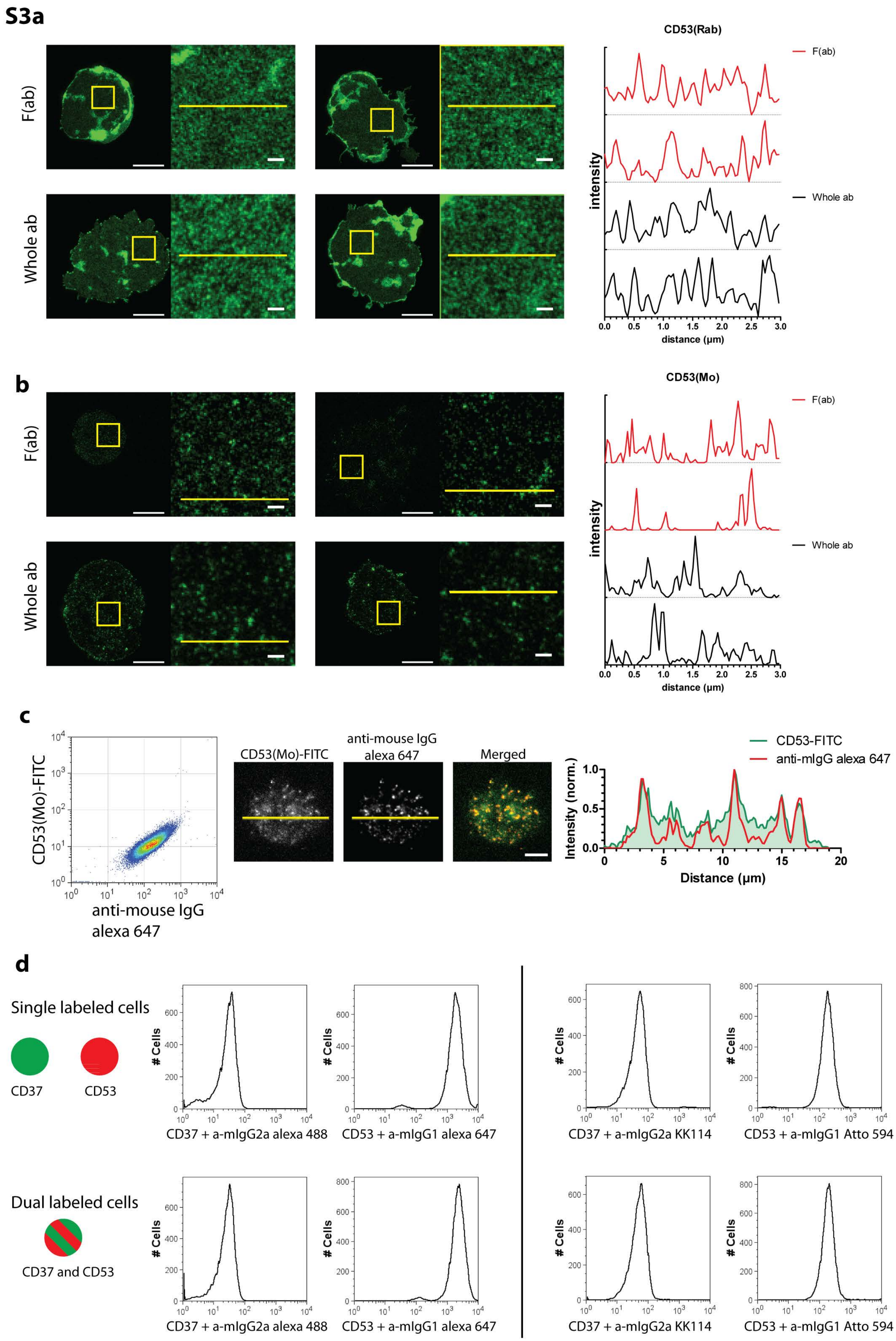
S3a
F(ab)
Whole ab
CD53(Rab)
F(ab)
Whole ab
intensity
distance (µm)
b
CD53(Mo)
F(ab)
Whole ab
intensity
distance (µm)
c
CD53(Mo)-FITC
anti-mouse IgG alexa 647
CD53(Mo)-FITC
anti-mouse IgG alexa 647
Merged
CD53-FITC
anti-mIgG alexa 647
Intensity (norm.)
Distance (µm)
d
Single labeled cells
CD37
CD53
Dual labeled cells
CD37 and CD53
Cells
CD37 + a-mIgG2a alexa 488
CD53 + a-mIgG1 alexa 647
CD37 + a-mIgG2a KK114
CD53 + a-mIgG1 Atto 594

S4

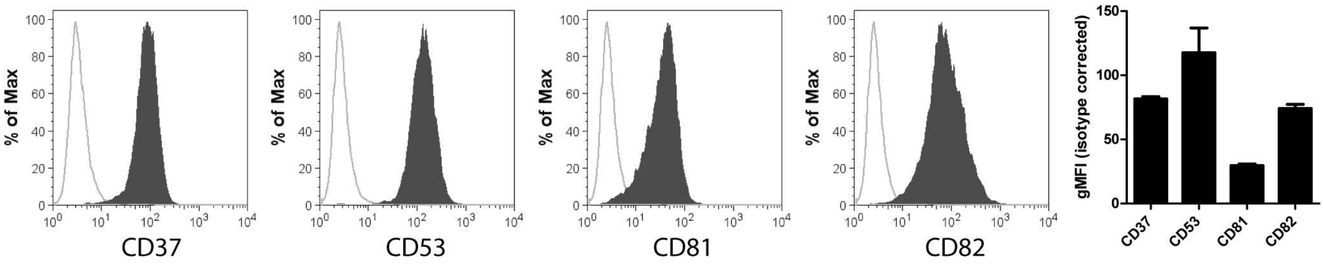

S5a

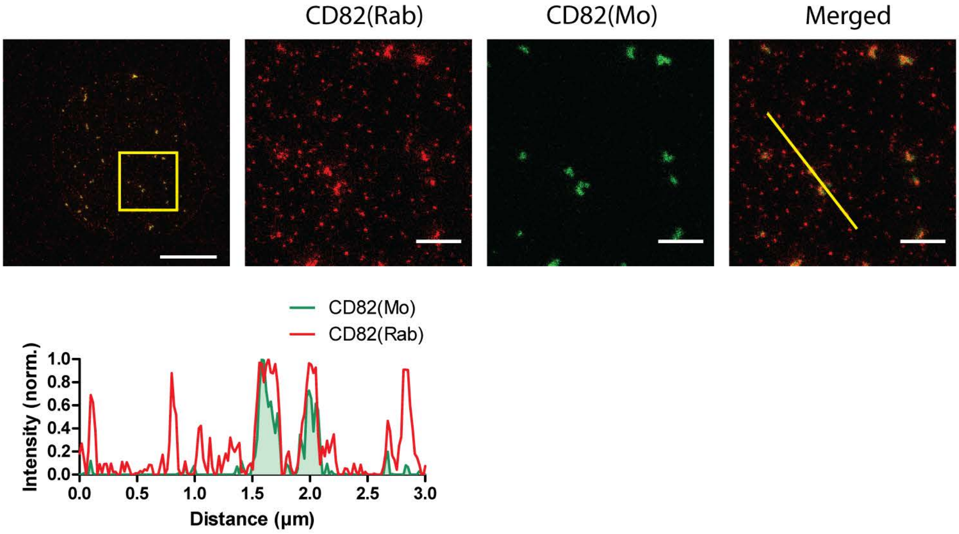
CD82(Rab)
CD82(Mo)
Merged
CD82(Mo)
CD82(Rab)
Intensity (norm.)
1.0
0.8
0.6
0.4
0.2
0.0
0.0
0.5
1.0
1.5
2.0
2.5
3.0
Distance (µm)

b

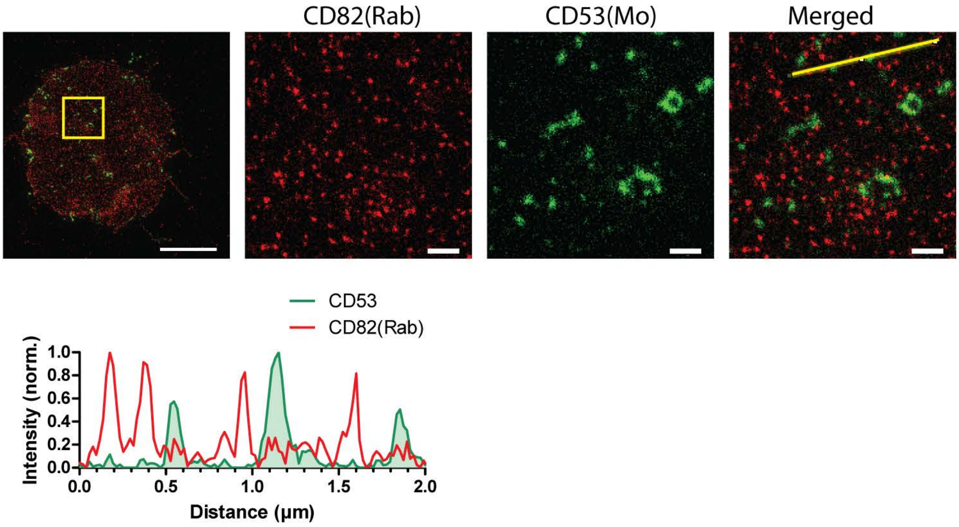
CD82(Rab)
CD53(Mo)
Merged
CD53
CD82(Rab)
Intensity (norm.)
1.0
0.8
0.6
0.4
0.2
0.0
0.0
0.5
1.0
1.5
2.0
Distance (µm)

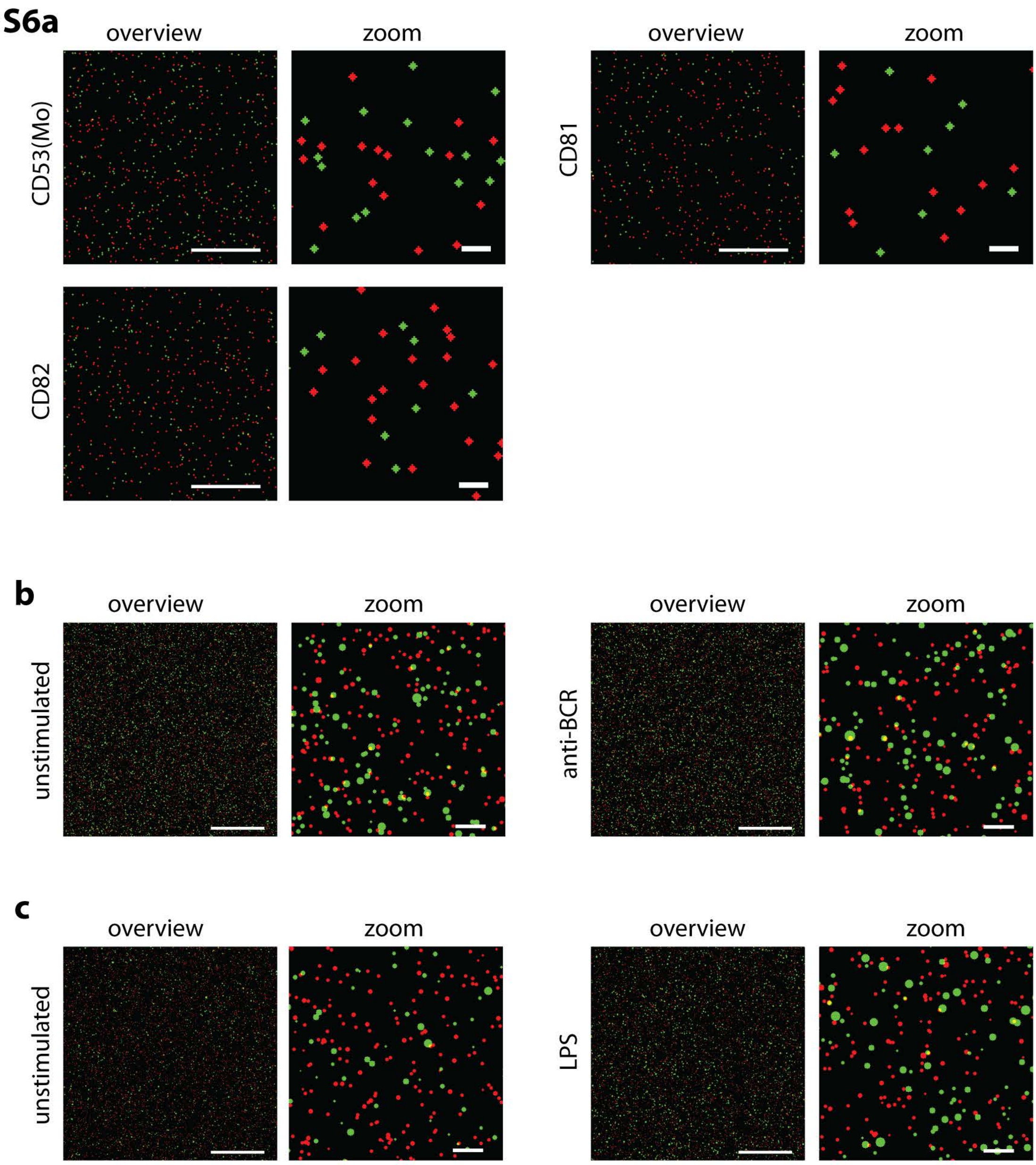
S6a
overview
zoom
CD53(Mo)
CD81
CD82
b
unstimulated
anti-BCR
c
unstimulated
LPS

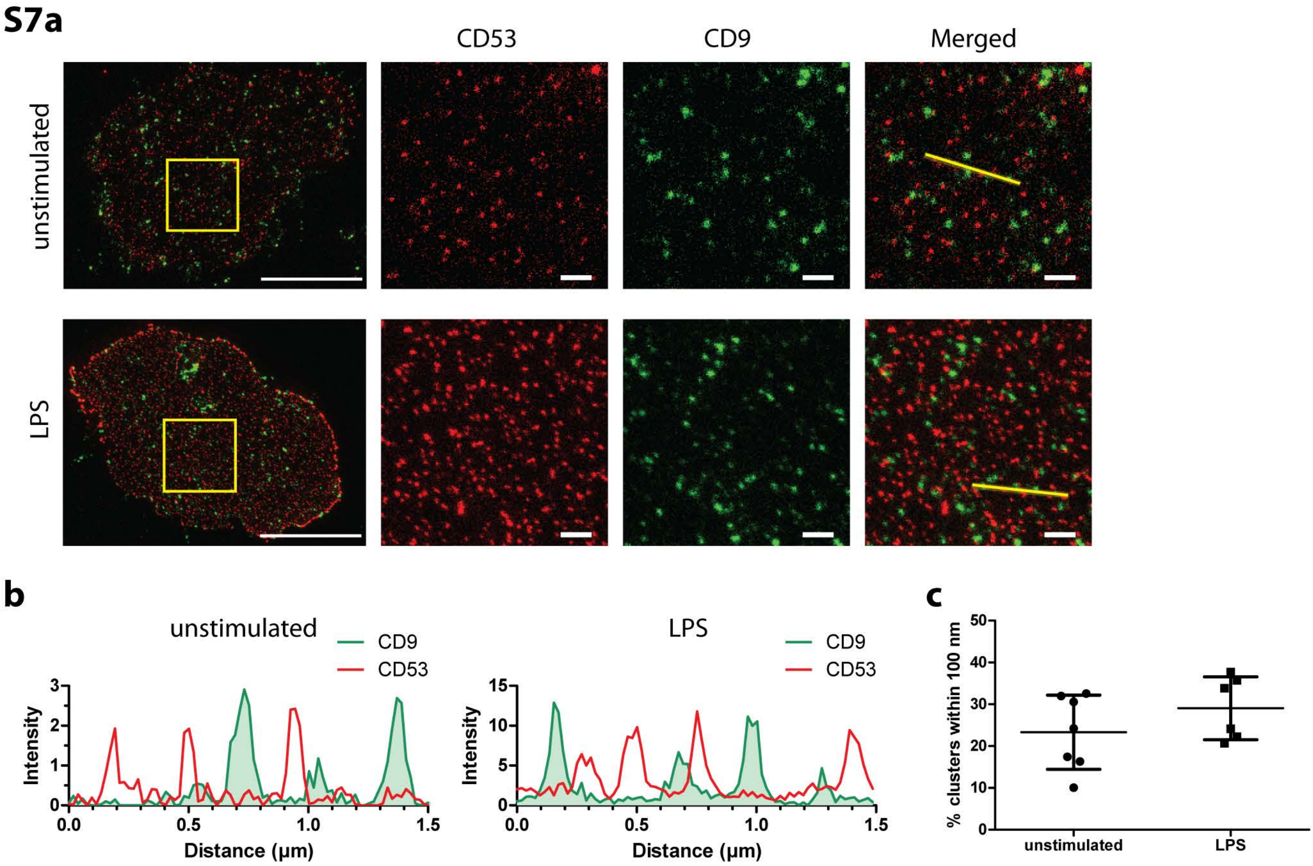
S7a
CD53
CD9
Merged
unstimulated
LPS
b
unstimulated
CD9
CD53
Intensity
Distance (µm)
LPS
CD9
CD53
Intensity
Distance (µm)
c
% clusters within 100 nm
unstimulated
LPS
